# Practitioners' validation of framework of team-oriented practice models in integrative health care: a mixed methods study

**DOI:** 10.1186/1472-6963-10-289

**Published:** 2010-10-14

**Authors:** Isabelle Gaboury, Heather Boon, Marja Verhoef, Mathieu Bujold, Laurent M Lapierre, David Moher

**Affiliations:** 1 Department of Community Health Sciences, University of Calgary, Calgary, Canada; 2 Leslie Dan Faculty of Pharmacy, University of Toronto, Toronto, Canada; 3 Faculté des sciences sociales, Université Laval, Québec, Canada; 4 Telfer School of Management, University of Ottawa, Ottawa, Canada; 5 Ottawa Methods Centre, Ottawa Hospital Research Institute, Ottawa, Canada

## Abstract

**Background:**

Biomedical and Complementary and Alternative Medicine (CAM) academic and clinical communities have yet to arrive at a common understanding of what Integrative healthcare (IHC) is and how it is practiced. The Models of Team Health Care Practice (MTHP) framework is a conceptual representation of seven possible practice models of health care within which teams of practitioners could elect to practice IHC, from an organizational perspective. The models range from parallel practice at one end to integrative practice at the other end. Models differ theoretically, based on a series of hypotheses. To date, this framework has not been empirically validated. This paper aims to test nine hypotheses in an attempt to validate the MTHP framework.

**Methods:**

Secondary analysis of two studies carried out by the same research team was conducted, using a mixed methods approach. Data were collected from both biomedical and CAM practitioners working in Canadian IHC clinics. The secondary analysis is based on 21 participants in the qualitative study and 87 in the quantitative study.

**Results:**

We identified three groups among the initial seven models in the MTHP framework. Differences between practitioners working in different practice models were found chiefly between those who thought that their clinics represented an integrative model, versus those who perceived their clinics to represent a parallel or consultative model. Of the scales used in the analysis, only the process of information sharing varied significantly across all three groups of models.

**Conclusions:**

The MTHP framework should be used with caution to guide the evaluation of the impact of team-oriented practice models on both subjective and objective outcomes of IHC. Groups of models may be more useful, because clinics may not "fit" under a single model when more than one model of collaboration occurs at a single site. The addition of a hypothesis regarding power relationships between practitioners should be considered. Further validation is required so that integrative practice models are well described with appropriate terminology, thus facilitating the work of health care practitioners, managers, policy makers and researchers.

## Background

Integrative healthcare (IHC) has become a popular term used to generally define healthcare practice that combines complementary and alternative medicine (CAM) and conventional treatments. Several definitions of IHC have been suggested since its inception in the late 1990s;[1-3] however, the academic and clinical communities have yet to arrive at a common understanding of what IHC is and how it is practiced [4,5]. The primary purpose of this paper is to assess the validity of a previously developed framework for categorizing team-oriented health care practice models.

The Models of Team Health Care Practice framework (MTHP) that emerged from an international workshop on the definition and operationalization of IHC, includes seven different models of IHC practice[6]. Each model is positioned along a continuum to represent the respective levels of integration of the biomedical and CAM paradigms, from a parallel to an integrative model of practice. A summary of the characteristics of each team-oriented practice model as described in the MTHP is presented in Table 1[6].

**Table 1:** Models of team health care practice in IHC

Model	Parallel	Consultative	Collaborative	Coordinated	Multidisciplinary	Interdisciplinary	Integrative
**Characteristics**	▪ Practitioners work in the same setting, but independently ▪ Roles are formally defined within one's clinical scope of practice	▪ Expert advice is shared between practitioners via personal contact, letter of referral note	▪ Patient is seen independently by each practitioner ▪ Practitioners informally share information concerning the treatment of a particular patient on a case-by-case basis	▪ Administrative structure stimulates collaboration ▪ Patients' files are shared between practitioners ▪ Liaison between practitioners is ensured by a manager/coordinator	▪ Leader in charge of the planning of patient care ▪ Each practitioner carries out treatment independently, according to his expertise ▪ Formalized extension of the coordinated model	▪ Planning of patient care is decided by a group of practitioners, via regular face-to-face meetings ▪ Extension of the multidisciplinary model	▪ Non-hierarchical holistic collaboration of practitioners ▪ Practitioners and patient contribute to patient care ▪ Extension of interdisciplinary model

**Group of models**	A		B				C

The models were developed around four key constructs of IHC from the practitioner's perspective: philosophy and values, structure, process, and outcomes. The philosophy and values construct defines how IHC is delivered to patients, from an epistemological perspective. In the framework, it is conceptualized as the guiding assumption underlying a practice model's structure, processes, and outcomes. The framework defines the structure construct by the types of individuals who make up the team, including the individuals' roles within the team as well as its infrastructure. Processes as defined by the framework identify how practitioners working in an IHC context relate to each other as well as the patient. Finally, outcomes are defined as the end products of the team activities (usually related to patient outcomes). This construct is the least well-developed in the original model [6]. The authors of the MTHP framework posited a series of hypotheses about how practice models differ based on the four key constructs of the framework. The MTHP framework, in particular the level of integration expected at the philosophical, structural, procedural, and outcome levels, has not yet been empirically tested. The objective of this study is to test nine of those hypotheses (MTHP hypotheses, Table 2), based on empirical data collected from practitioners working in a Canadian IHC context.

**Table 2:** Constructs distribution across models of team health care practice

MTHP hypothesis As integration increases... (i.e. Moving along the continuum from Group A to Group C models...)	Scale*	Group A Median (IQR)	Group B Median (IQR)	Group C Median (IQR)	p-value	Hypothesis supported (quantitative results)	Hypothesis supported (qualitative results)
Diversity of health care philosophy and involvement of each team member increase (Philosophy and values 1)	Beliefs in benefits of interprofessional collaboration	4.7 (3.9, 5)	5 (4.6, 5)	5 (4.9, 5)	0.029	✓	✓
Reliance on the biomedical model decreases (Philosophy and values 2)	Knowledge of each other's healing approach	14 (11.8, 15)	14 (12, 15)	15 (12, 15)	0.889		✓

Trust and respect among members increase (Structure 1)	Trust	21 (19, 24)	24 (22, 25)	25 (22.3, 25)	0.034	✓	N/A
Complexity of the organizational structure of the clinic increases (Structure 2)							✓
Presence of hierarchical links and defined roles between practitioners decrease (Structure 3)						N/A	

The communication process increases (Process 1)	Knowledge donating	3.8 (3.5, 4.2)	4.3 (3.8, 4.8)	4.8 (4.1, 5.0)	< 0.001	✓	
	Knowledge collecting	4 (3.7, 4.7)	4.7 (4, 5)	5 (4.3, 5)	0.014	✓	
Practitioner autonomy decreases (Process 2)	Physician centrality	10.5 (9.8, 14)	8 (5, 12)	8 (3, 12)	0.169		✓
Respect for diversity of opinions and importance for consensus-based decisions increases (Process 3)	Conflict associated with interprofessional collaboration	2.6 (1.3, 3.1)	1.4 (1, 1.8)	1.4 (1, 2)	0.030	✓	N/A

Complexity and diversity of the outcomes increase (Outcomes 1)	Satisfaction	4.7 (3.8, 5)	4.8 (4.3, 5)	5 (5, 5)	0.015	✓	
	Personal growth	4.6 (4, 5)	4.4 (4.1, 4.8)	5 (4.5, 5)	0.002	✓	✓
	Intention to leave in the following year	1.3 (1, 2.3)	1.3 (1, 2)	1 (1, 1)	0.040		

* Scores were calculated from the average of scale items from a negative anchor (1) to a positive anchor (5) or from the total score of the items from the same anchors. The sum of the items is presented for the Physician centrality scale from a negative anchor (0) to a positive anchor (4) for a maximum score of 24. The maximum score for the Knowledge of each other's healing approach was 15.

N/A = not available

In Canada, most of the IHC clinics recover the physicians' fees throughout the public healthcare system, which offers universal reimbursement. CAM practitioners, on the other hand, are paid by directly by patients and/or insurance companies. Very few clinics propose packages to their clients within which physician fees are covered. Moreover, the regulation of CAM practitioners varies from one province to another, which has been found to impact relationships between practitioners, especially between biomedical and CAM practitioners, but also among CAM practitioners [7,8].

## Methods

We conducted a secondary analysis using data sets of two original research projects (one qualitative and one quantitative) that focused on interprofessional collaboration within Canadian IHC clinics. Quantitative and qualitative methods were used to examine overlapping and distinct facets (philosophy and values, structure, process, and outcomes) of a phenomenon (integrative health care) and seek convergence of the results[9]. Convergence of the results is confirmed when results of both qualitative and quantitative inquiries results support a hypothesis of the MTHP framework, i.e. when the results are consistent across methodologies.

The first data set consists of verbatim data of qualitative interviews with practitioners working in Canadian IHC clinics, operationally defined as clinics where at least three different practitioners work in the same location. The teams had to include at least one physician licensed to practice in Canada and CAM practitioners including (but not limited to): naturopathy, massage therapy, chiropractic, and Traditional Chinese Medicine, including acupuncture. Practitioners, other than physicians, with a biomedical health care background, such as nurse practitioners, physiotherapists, or pharmacists were also eligible. The interviews explored practitioners' experiences of interprofessional collaboration in their own setting. The second data set consisted of data from a quantitative survey of Canadian IHC clinics' practitioners which measured key items (e.g. education, trust, job satisfaction) related to interprofessional collaboration. Each research project is summarized below.

### Research Project I: Face-to-face interviews

The primary objective of Project I was to describe how IHC is experienced within Canadian IHC clinics. Project I drew upon the Input Process Output model, a comprehensive and tested model that has helped significantly to shape the literature on teams, in terms of capturing themes illustrating the interplay between and amongst individual team members and the team as a single functional entity [10]. Purposeful sampling was used to ensure a wide range of IHC clinics across Canada. Additionally, maximum variation sampling of practitioners was used to ensure a broad representation of practitioner's expertise. Interviews were conducted using a series of semi-structured open-ended questions. Twenty-one practitioners from 5 different IHC clinics in 4 different provinces were interviewed. Almost half of them (n = 10) were primarily biomedical practitioners. Further details of the project are reported elsewhere [8].

### Research Project II: Survey

In Project II, the Relationship-centered care model was used as a guide to explore the relationship between the key constructs of interprofessional collaboration from practitioners working in an IHC clinic [11]. Snowball sampling using various key informants such as web sites, news groups, professional associations and conferences attendees was used to compile a list of Canadian IHC clinics until saturation of the list was obtained. All practitioners were contacted by mail to complete a quantitative survey. Face validity of the survey scales was assessed and all but one (Knowledge of each other's healing approach) were measured using scales that had previously been reported to be reliable and valid. Forty-three biomedical (e.g., physicians, nurses, and psychologists) and 42 CAM practitioners out of 218 potential respondents returned their completed questionnaires. These practitioners belonged to 25 clinics. Two practitioners did not identify their medical background (biomedical or CAM). Further details on the quantitative survey and its questionnaire development can be found elsewhere[12]. A description of the scales selected for this paper is provided in Table 3.

**Table 3:** Quantitative scales

Framework construct Survey scale	Description	Cronbach's α for this paper
**Philosophy**		
Believes in benefits of interprofessional collaboration	Extent to which practitioners surveyed see strengths in multidisciplinary collaboration, both from a patient and practitioner' perspective (5 items) [19]	0.882
Knowledge of each other's healing approach*	Respondent's openness and awareness to working with professionals of a different healthcare paradigm - combination of items from [20] and qualitative work of (Gaboury, 2009). (3 items)	0.824
**Structure**		
Trust	Extent to which an individual is willing to trust and have confidence in the words and actions of his colleagues (5 items) [21]	0.911
**Process**		
Knowledge exchange	Respondent's perception of the extent to which knowledge is shared between the individuals of a given environment using the Knowledge donating (KD) and Knowledge collecting (KC) sub-scales (8 items) [22].	0.815 (KD) 0.751 (KC)
Physician centrality	Level of acceptance of sharing of authority and leadership among the members of the team (6 items) [23]	0.682
Conflict associated with interprofessional collaboration	Extent to which issues related to interdisciplinary collaboration (such as competition and sharing of responsibilities) could lead to conflicts within a healthcare clinic (8 items) [19]	0.886
**Outputs**		
Satisfaction	Overall satisfaction with work via a 3-item scale [24]	0.713
Personal growth	Respondent's perception of the opportunities for achievement and challenge in their working milieu (4 items) [25]	0.792
Intention to leave in the following year	3-item scale created by Bishop and colleagues [26]	0.787

* Resulting scale not formally validated.

Research ethics approval for the project was obtained from the Research Ethics board of the University of Ottawa and the Children's Hospital of Eastern Ontario prior to contacting practitioners for both original studies.

### Data analysis

Qualitative data were analyzed by the interviewer using NVivo 7.0 and basic content analysis [13]. To address small sample issues in Project II, the 7 original models in the framework were merged into 3 groups, labelled A, B, and C. Grouping of the models was based on conceptual similarities between models [6]. The first two models on the continuum (parallel and consultative) were grouped based on the minimal communication and personal interactions between the practitioners - group A. The collaborative, coordinated, multidisciplinary and interdisciplinary models were grouped based on the fact that exchanges between practitioners are expected and encouraged by the models' philosophy and values, as well as structure - group B. Finally, the integrative model forms the third group since fully integrative practice is an important extension of the continuum of team-oriented practice models - group C.

Prior to the qualitative analysis, the lead author assigned each clinic to one group (A, B or C) that best represented the clinics. This decision was made based on field observations and discussions with the staff. Key themes of the analysis were defined according to the constructs and underlying hypotheses arising from the continuum of the MTHP framework. All data were reread several times to reveal the excerpts that illustrated the key themes. As a quality control check, codes for 3 randomly selected interviews were independently extracted by a second member of the team, who also thoroughly reviewed the results. In cases of disagreement, consensus was reached between the two analysts through discussion.

As part of the original quantitative data collection, the respondents were asked to select a model from the MTHP framework that best represented the collaboration model at the clinic in which they worked. Respondents were blinded to the models' names to ensure they did not select models because of their names alone. Models were displayed in a table similar to Table 1 except the models' names were removed. Results were summarized using descriptive statistics including frequencies and median (interquartiles range [IQR]). With the exception of physician centrality, intent to leave current employment and level of conflict scales, a higher score represents a positive outcome. Given the skewness of the distribution observed in many of the survey constructs, a non-parametric approach (Kruskal-Wallis test) was used for comparisons across the 3 practice model groups. *Post-hoc *analyses using the Conover method were conducted to compare models in a pairwise fashion [14]. P-values are two-sided and were declared statistically significant when they reached a 0.05 probability level. For respondents who chose not to answer the model question or circled more than one model, the health care practice model was imputed by using the model that was most commonly selected by the other respondents working in the same clinic. Sensitivity analyses were conducted with and without those respondents.

## Results

Health care practitioners from 25 different IHC Canadian clinics, four of which also took part in the qualitative phase of the project (the fifth clinic that was involved in Project I closed prior to the administration of the survey). The distribution of the responding clinics across the different MTHP groups of models as selected by the respondents is presented in Table 4. Overall statistical comparisons of the models according to the MTHP framework constructs (philosophy and values, structure, process, and outcomes) are presented in Table 2. Pairwise comparisons between groups of models as well as qualitative findings are discussed in the sections below.

**Table 4:** Distribution of clinic models as perceived by the survey respondents

	Models				
**Clinic number**	**Group A**	**Group B**	**Group C**	**Number of respondents who did not choose a model**	**Total number of respondents**

1	0	0	5	1	6
2	1	0	0	0	1
3	0	0	3	1	4
4	1	1	0	0	2
5	0	0	1	1	2
6	0	1	0	0	1
7	1	1	1	0	3
8	1	2	5	1	9
9	1	2	0	0	3
10	0	1	1	0	2
11	0	3	4	0	7
12	1	1	3	0	5
13	0	1	2	0	3
14	0	3	0	0	3
15	0	1	0	0	1
16	1	0	4	0	5
17	0	0	1	1	2
18	1	0	0	0	1
19	0	0	2	0	2
20	0	1	1	0	2
21	1	0	1	0	2
22	1	2	9	0	12
23	0	2	2	1	5
24	0	1	0	0	1
25	2	1	0	0	3
Total	12	24	45	6	87

### Philosophy

The survey measured the extent to which the practitioners believed in the benefits of interprofessional collaboration. This was used to verify whether diversity of health care philosophy and involvement of each team member increase when moving along the continuum (hypothesis *Philosophy and values 1*). This hypothesis was supported as respondents identifying themselves as being from group C clinics scored significantly higher on the beliefs in benefits measurement scale than the respondents from the group A models (p = 0.031). However, the overall difference was no longer significant when the respondents who chose not to pick a model of practice were added to the sample (p = 0.094). Similarly, no statistically significant difference was found between groups in the respondents' perception of their knowledge of each other's healing approach (p = 0.889).

Qualitative analysis of the data also revealed differences between models of practices in terms of the philosophy of the interviewees towards IHC, especially with regards to how they defined the practice and envisioned the place of IHC in the current Canadian health care system. In contrast to interviewees who classified their collaboration model as group A, a majority of the participants from more highly integrated practices (group B or C) emphasized that they were practicing health care according to a patient-centered approach where the multiple facets of wellness were considered, a notion that the former group did not bring up in discussions.

*"Oftentimes your standard medical conventional treatment for certain ailments will treat the symptoms but, peripheral things [also] need to be addressed. [...] I think our current health care system is overwhelmed and I think that a lot of times we're doing damage control, but not in this clinic. I feel that the patients that walk into this clinic get the most cutting-edge care you can get because you get both sides of the coin without any animosity and that's what needs to happen for our medical system to progress. The doctors and naturopaths and osteopaths and chiropractors need to put their egos aside and work together for the betterment of the patients because that's what it's about." *(Multidisciplinary clinic, Complementary and Alternative Medical practitioner [CAM1])

Similarly, the director of a group C clinic described his model of practice as follows: "[*Our model is*] *effective and more real than the conventional model and is more attuned to complex multi-factorial chronic symptoms. [...] Every patient gets 5 acupuncture sessions, everybody gets 3 hellerwork sessions, everybody gets 6 counselling sessions and so on. What we do within those sessions is unique to the individual*. (Integrative clinic, Biomedical practitioner [BM1])

Our results validated the second hypothesis pertaining to the Philosophy and values set on the reliance on a biomedical model. For example, in many instances participants in a group C model pointed out that their clinics were not offering two different kinds of medical health care services, but rather blended approaches to health care where the practitioners serve as guides for the patient's healing process.

Furthermore, we found that the terminology related to "evidence-based medicine", a concept often associated with the biomedical scientific culture, was common in the discussion with the interviewees from clinics similar to the group A models, but specifically from the group B models. These participants alluded to the fact that evidence-based medicine was central to the clinic's ethos. Some CAM interviewees associated an evidence-based clinical approach with rigidity and criticized it either as a limitation for their own practice or likened it to shackles for their conventional medicine colleagues. However, the interviewees in group C seemed to have a more open-minded view of evidence-based practice:

*The important thing is that [the staff] have reasonable training in conventional work so they are not kind of wishy washy, New age-type-grounded, but that they also have to explore their own healing, for one reason or another, because it is only through the experience of process or finding their own healing that you ever understand the holistic perspective. [...] The only kind of learning you can do in regular medicine is to see something you have not seen before. What you learn [in integrative medicine] is to get rid of everything you ever learned. The only thing you learn is that everything you ever learned needs to be trashed. *(Integrative clinic, BM1)

### Structure

The first hypothesis referring to the structure construct corresponds to trust and respect among the team members (hypothesis *Structure 1*). Interpersonal trust between colleagues was found to be the lowest within respondents from the group A models when compared both to those from group B (p = 0.045) and to those from group C (p = 0.014). No significant difference was found between groups B and C (p = 0.720).

Different perspectives on structure emerged from the qualitative data. The relative simplicity of the structure of the visited clinic identified as collaborative was striking. As one interviewee expressed it:

*"We are 4 people sharing the expenses of one centre, that's all. We share the business expenses, we share the rent, other than that we do not have any dependency. I don't even know when they come and when they go. [...] We do not work for each other." *(Consultative clinic, BM2)

In contrast, participants from a group C model described the barriers met with regards to the clinic management of space and time (hypothesis *Structure 2*) as well as sustainability of the particular health care services offered to the clientele:

*"We have a nutritionist on staff. If we do not refer to our nutritionist then she is sitting there in a room not making any money for the clinic and nothing works. So paying for the rent or the space and being a not-for-profit clinic, we have to make sure that we do not go under budget and just break even." *(Interdisciplinary clinic, CAM1)

*"I think [working within a collaborative setting] is more time consuming, because you basically increase your load of patient care since you not only work a full day of patients, you also possibly help with other patients. *(Interdisciplinary clinic, CAM2)

With regards to the business plans of the different clinics approached for Project I, we also noted contrasting methods of reimbursement for the delivery of care. In the single consultative clinic (group A), CAM practitioners saw their fees reimbursed from patients' insurance plans and personal payments; whereas the physicians only invoiced the government for their services. In the three clinics corresponding to group B of the MTHP framework, we observed a mixture of reimbursement methods within each clinic. Even physicians sometimes invoiced the patients directly, especially when the care provided was not considered conventional medicine. In the group C clinic, all practitioners were paid directly by the patients or their insurance plans, whenever possible.

The clinical hierarchical structure (hypothesis *Structure 3*) was not found to differ between models as suggested by the framework and therefore, this hypothesis could not be supported by our data. For example, group B models were the only ones with either a clinical (biomedical) director or a board of chief executive officers. However, when this topic was discussed, all interviewees confirmed that this structure was in place for management purposes and did not interfere with clinical decision making. The two clinics in groups A and C included in the interviews did not have any formal chain of command in place. However, they were the two smallest clinics, with 6 staff members each, compared with a range of 8 to 22 in the other three group B models.

### Process

The survey results verified that knowledge sharing is closely related to the MTHP continuum (hypothesis *Process 1 *on communication within the team). The manner in which information and knowledge is gathered and then shared among practitioners was measured using two different scales. Information sharing was found to vary between clinics according to each pair of models (p = 0.017 between groups A and B; p < 0.001 between groups A and C; and p = 0.035 between groups B and C) in the direction expected according to the framework. The second measure, knowledge gathering, was found to differ significantly only between groups A and C (p = 0.006).

The degree of acceptance of sharing authority amongst the members of the team, and particularly the clinical autonomy of the biomedical practitioners of the team compared with the CAM practitioners (hypothesis *Process 2*) was not found to differ significantly between groups of models (p = 0.169). However, this was only one facet of practitioner autonomy. Independence in clinical decision making was found to vary slightly among the models. From the consultative clinic visited (group A), the independency of practitioners was confirmed in many instances during the interviews: *"We all do our job and that's it. We don't have anything in common." *(Consultative clinic, BM1). Additionally, we found that reliance on colleagues' opinion or services between the biomedical and CAM practitioners of the consultative clinic was almost nonexistent, which demonstrated important autonomy of the practitioners but little opportunity for synergy and building of trust within the team members.

In contrast, compromising was common in daily practice for a few interviewees working in other clinic models. *"For the practicality of the program, the decision might be more specific, hormone first before getting to the other stuff. So, if that was really important to me I might be upset, right? And it gets back to the whole ego and working as a group. It is just putting aside what I would normally do or see as a priority. The group would have to come together; everyone has to make that compromise." *(Interdisciplinary clinic, CAM3). However, this type of comment was not found to be consistent across the interviewees as most of them considered themselves independent of their colleagues. The data suggested that the loss of autonomy would be for the sake of better answering the patients' needs:

*"I think the main thing is that it's the client who does the healing, we don't. It's helping the client explore what they need to explore. It's not a case of "This is the right way to do it." We would certainly share with each other things like, "This approach might work better with this person." But it's not having a lot of, I guess investment in being right about how it should be done. I guess our main thing, in a way, is trying to support people to make it a safe enough place for them to do the degree of letting go and exploring that thing." *(Integrative clinic, CAM1)

Finally, we found that the level of conflict associated with collaboration, which we used as a proxy for the respect for the diversity of opinions among team members (hypothesis *Process 3*), was correlated along the continuum of models. In fact, conflicts within the team members seemed to occur more frequently within the group A models when compared to the group B models (p = 0.017), or to group C models (p = 0.012).

### Outcomes

The purpose of the MTHP framework was to delineate various models of practice that could be useful for patients and practitioners. Hence, the theoretical hypotheses related to the outcome construct focused mainly on patient health outcomes and the cost-effectiveness of care. Projects I and II did not include any of the measures related to health care or financial effectiveness of the clinics.

An important outcome from a practitioner perspective, job satisfaction, was higher for the respondents identifying themselves with group C models compared to the group A models (p = 0.050). These later respondents also reported seeing significantly less opportunity for personal growth compared to the respondents in group C (p = 0.005). These findings did not completely agree with the qualitative data since the vast majority of interviewees reported being quite satisfied with their job regardless of the clinic model assigned for the purpose of this project. Practitioners in group C described their working environment to be challenging, less stressful and frustrating, and more collegial, friendly and healthy than other health care delivery settings they have or had worked in. On the other hand, we noted that the interviewees working in a clinic in group A were less explicit in terms of the benefits of interprofessional collaboration for themselves compared to the other interviewees; rather they tended to refer more often to the positive aspects of their collaborative practice from a patients' perspective.

When asked about their intent to leave the clinic in the next year, respondents who selected a group A model to represent their clinic were not as homogeneous in their intent to stay as the other respondents. Although we observed a significant difference between groups (p = 0.040), it did not remain significant when the model was imputed for the respondents who did not chose a particular model of practice (p = 0.074). Additionally, no significant differences could be found between groups when compared in a pairwise fashion. No conflicting findings were found from analysis of the interviewees' discourse. In fact, when this theme was discussed, most interviewees emphasized that this current position compared advantageously in many respects with other health care practice models they had worked within, currently or in the past.

## Discussion

The main aim of this project was to test hypotheses relating to the continuum of team health care practice models. The triangulation of quantitative and qualitative methodologies was valuable to analyse a broader array of characteristics meant to illustrate the differences between clinical models of practice around four key constructs of IHC: philosophy, structure, process and outcomes. The convergence of the results of the two methodologies strengthened the credibility and applicability of the framework. From an organizational perspective, respondents who identified themselves with a parallel or consultative practice model (group A) differed significantly from practitioners in the other practice models proposed by Boon and colleagues' continuum with respect to most of the hypotheses inherent in the key constructs of the framework. Respondents who selected models in groups B or C were found to be more comparable for most of the framework's constructs, with the exception of information sharing process which was happening more frequently within the integrative practice models (group C). This suggests that fewer than seven different models might be occurring in the IHC field in Canada and that differences between those models are likely weaker than what the framework proposed.

We were careful not to present the labels of the clinical models in the survey questionnaire, so that the respondents would associate the clinic's practice model with the definition given and not the label itself. One intriguing result was a lack of agreement between practitioners within clinics in terms of the practice model under which the team evolved. There are three possible explanations for this. First, within an IHC clinic, different practice models may apply to interactions among various pairs of practitioners due to personal compatibility, paradigm complementarity or simply scheduling details that allow or preclude personal interactions. Second, the practice of IHC suggests individualization of patient care and thus, some patients may choose to access only one or a limited number of practitioners in the clinic, thereby modifying the collaborative patterns among the team members. Third, we cannot exclude the possibility that the description of each practice model is not specific enough to allow practitioners to select a model that most accurately represented their practice. For example, we found that an unexpectedly high number of respondents picked the integrative model (group C) to describe their practice. This could be because the description of this specific model was not sufficiently precise compared to descriptions of other models of the framework, or a social desirability bias in that the participants saw this description as the "best" way to practice. Further validation of the specificities of the practice models' descriptions is required. *In-situ *case studies of different models classified *a priori *according to the framework would allow a better assessment of subtle differences between clinics as well as refinement of the description of each model. Nonetheless, we believe that the conclusions of this project are likely to remain valid, even in the eventuality that the integrative practice model description needs refinement, because most of the differences were found between the group A models and the rest of the continuum's practice models. We assumed that sufficient details were given to the survey respondents concerning each model so that the integrative model would not be mistakenly selected in place of the parallel or consultative one (group A) if the former model of collaboration was truly occurring between the respondent and the rest of the team.

Organizational theory differentiates between the hierarchical structure of a team and the power relationships among the team members [11]. The team structure explicitly defines the roles of the members and outlines the chain of command as in an organizational chart, if such a hierarchy exists. However, in practice, team members may interact with each other according to implicit rules not necessarily laid out in such a bureaucratic fashion. For example, dominance by a biomedical practitioner within a team where the leadership is, on paper, shared between health care providers is an example of a flat hierarchical team structure where power relationships are important to examine. A limitation of the framework was that it does not include any hypothesis on power relationships among the team members. In this project, we were not able to demonstrate any particular impact of the hierarchical organization of the clinics on the process of collaboration. As discussed by Block, our findings supported the hypothesis that the presence of a structural hierarchy within the clinic is less likely to impact the integration of health care as clinical authority and influence are dissociated from the formal organizational structure of the clinic[4]. In our sample, the interviews revealed that such a structure was often in place for administrative purposes rather than clinical decision making. In fact, none of the participants interviewed were able to identify a formal leader in the role of a clinical decision maker because those decisions were largely dependent on the patient case rather than the practitioners' role(s). However, since conflicts associated with a collaborative process are occurring within the clinics, as shown by our quantitative data, we could possibly relate them to power relationships, which had been found elsewhere to be a key component of the process of collaboration within Canadian IHC clinics [7,8]. Thus, we propose a hypothesis that power relationships are expected to be less apparent as one moves along the continuum towards increased integration of health care practices. This addition emphasizes that the differences are likely to be observed in the process of collaboration rather than the structure of the clinic, as currently suggested by the framework.

It is important to note that the purpose of Boon and colleagues' continuum was not to determine the ideal model of care, but rather to identify that different models of care exist and to facilitate explorations of questions about what models of care are most appropriate in specific contexts or patient populations. In this work we also embraced that philosophy. Boon and colleagues clearly recognized that the optimal model of practice should be determined by both the patient's needs and the interpersonal styles and professional requirements of the practitioners involved in the team. On this note, we found that caution must be taken when using the framework to label the type of collaboration occurring within a clinic since the model might vary depending on the patient's choice, the patient's condition, the dyad of practitioners, and the practitioner's personal and professional experience.

### Implications

The validation of this conceptual framework has implications for IHC practitioners as well as for patients. Often times researchers assume that teamwork is a one-dimensional concept and study its impact on healthcare, assuming that all teams will provide similar results. Our study suggests that teams appeared to differ in terms of their philosophy and values, structure, processes, and outcomes, depending on the model of collaboration underlying the clinic or dictated by specific patient needs and desires. This study is a key step forward in clarifying differences between team-oriented practice models in an IHC setting. A uniform and broadly recognized understanding of the many types of models in IHC will make comparison and evaluation of models easier and will improve the communication within and among practitioners, researchers, and, most importantly, patients.

### Study limitations

The relatively poor response rate at the participant level may limit the validity of the quantitative results. We attempted to survey the entire population of practitioners working in Canadian IHC clinics, and note that most of the non-respondents were from very large clinics with a majority of practitioners working under a parallel and consultative practice model (assessed throughout an introductory discussion of the project with the clinic's managers). Assuming that the respondents from group A are those who are more likely to collaborate and to believe in the benefits of IHC, in reality the differences between practice models are likely to be greater than those actually observed in this project.

Additionally, the response rate might have limited our capacity to find differences between groups B and C, due to a lack of statistical power. However, our response rate is similar to the physician response rates to other surveys reported in the literature [15-17]. In addition, research has shown that nonresponse bias may be of less concern in physician surveys than in surveys of the general public because demographic variables have not be found to differ between respondents and non-respondents. Moreover, the same paper found that physicians tend to represent a more homogeneous group with respect to knowledge, training, attitudes, and behavior when compared to the general public, which suggest that respondents are likely to be representative of the whole population of physicians [17].

The qualitative results suffered from the limitations inherent to a secondary analysis[18]. Namely, saturation of the themes related to the characteristics of the framework was not systematically reached within each group of practice models. However, we believe that this limitation was countered by the richness of the information gathered, as well as the convergence obtained through data triangulation. Additionally, participants were not purposefully selected based on the practice model they are involved with and the possibility of our results not showing differences between practice models where in fact differences exist is not excluded. Consequently, changes along the continuum between models that could not be validated with this project may well emerge with a different sample of clinics.

## Conclusions

Our results supported most of the hypotheses underlying the continuum of the MTHP framework and showed that the most striking differences lay between the parallel and consultative models (group A) and the five other models along the framework (groups B and C). Additional work is needed to assess whether as many as 7 distinct models of collaboration truly occur, especially since our evaluation could often not detect major differences between models in groups B and C. Collapsing the 7 models into the 3 used in this work appears to reflect better the Canadian reality and would be more practical for research purposes. The findings suggest that such a framework should be used with caution to guide the evaluation of the impact of each model on both subjective and objective outcomes of IHC such as patients' functional status, and quality and cost-effectiveness of care, because more than one model of collaboration could take place within a single clinic. On that note, an important voice missing in this project is that of the patients themselves, which was beyond the objectives of this project. The conceptual framework suggested that patients' roles change across the continuum of practice models, but this could not be verified with the data collected. Additionally, further validation is required to ensure that the description of the integrative practice model is well understood and that terminology is used appropriately and with a common understanding by health care practitioners, health care managers, policy makers and researchers.

## Competing interests

The authors declare that they have no competing interests.

## Authors' contributions

All authors were involved in the design of the study and conduct of the study. IG and MB performed the analysis of the qualitative data. IG performed the analysis of the quantitative data. The qualitative analysis has been reviewed by DM and HB. IG prepared a first draft of the manuscript. All authors read and approved the final manuscript.

## Pre-publication history

The pre-publication history for this paper can be accessed here:

http://www.biomedcentral.com/1472-6963/10/289/prepub
